# Preliminary evidence for a change in spectral sensitivity of the circadian system at night

**DOI:** 10.1186/1740-3391-3-14

**Published:** 2005-12-11

**Authors:** Mariana G Figueiro, John D Bullough, Robert H Parsons, Mark S Rea

**Affiliations:** 1 Lighting Research Center, Rensselaer Polytechnic Institute, 21 Union Street, Troy, NY 12180, USA; 2 Department of Biology, Rensselaer Polytechnic Institute, 110 8th Street, Troy, NY 12180, USA

## Abstract

**Background:**

It is well established that the absolute sensitivity of the suprachiasmatic nucleus to photic stimulation received through the retino-hypothalamic tract changes throughout the 24-hour day. It is also believed that a combination of classical photoreceptors (rods and cones) and melanopsin-containing retinal ganglion cells participate in circadian phototransduction, with a spectral sensitivity peaking between 440 and 500 nm. It is still unknown, however, whether the spectral sensitivity of the circadian system also changes throughout the solar day. Reported here is a new study that was designed to determine whether the spectral sensitivity of the circadian retinal phototransduction mechanism, measured through melatonin suppression and iris constriction, varies at night.

**Methods:**

Human adult males were exposed to a high-pressure mercury lamp [450 lux (170 μW/cm^2^) at the cornea] and an array of blue light emitting diodes [18 lux (29 μW/cm^2^) at the cornea] during two nighttime experimental sessions. Both melatonin suppression and iris constriction were measured during and after a one-hour light exposure just after midnight and just before dawn.

**Results:**

An increase in the percentage of melatonin suppression and an increase in pupil constriction for the mercury source relative to the blue light source at night were found, suggesting a temporal change in the contribution of photoreceptor mechanisms leading to melatonin suppression and, possibly, iris constriction by light in humans.

**Conclusion:**

The preliminary data presented here suggest a change in the spectral sensitivity of circadian phototransduction mechanisms at two different times of the night. These findings are hypothesized to be the result of a change in the sensitivity of the melanopsin-expressing retinal ganglion cells to light during the night.

## Background

It is well established that the absolute sensitivity of the suprachiasmatic nucleus (SCN) to photic stimulation received through the retino-hypothalamic tract (RHT) changes along the 24-hour day [1-4]. Conceivably, changes in the sensitivity of the circadian system to light/dark patterns could be driven by the master clock in the SCN, by a peripheral clock in the retina, or by both.

Jagota et al. [4] showed that neural activity in the hamster SCN varied over the 24-hour cycle, suggesting the existence of a morning and an evening oscillator in the SCN. Changes in photoperiod affected the two SCN peak activity periods differently, demonstrating that the phases of the two peaks are not locked but are independently linked to the environmental cycle of dusk and dawn. Moreover, they showed that the two peaks responded differently to a pulse of glutamate (the neurotransmitter that conveys light information from the eye to the SCN). Glutamate, when given after dusk, delayed the evening peak but not the morning peak; when glutamate was given before dawn, the early peak was advanced but the evening peak was unaffected. Pevet et al. [1] also demonstrated that the duration of the SCN phase sensitivity to light is closely related to the length of the night. The SCN phase sensitivity to light was measured in terms of the expression of *Fos *protein, which is considered a marker of SCN cell response to light stimuli. The findings of Jagota et al. [4] and of Pevet et al. [1] reinforce the growing evidence for temporal changes in the SCN's sensitivity to light. Unknown, however, is whether there is temporal variation in the sensitivity of the circadian phototransduction mechanism itself throughout the 24-hour cycle.

Lucas et al. [5] have shown that light can reset the circadian clock as well as stimulate the iris light reflex of genetically-manipulated mice without classical photoreceptors (rods and cones). Berson et al. [6] showed that a subset of retinal ganglion cells (RGCs) innervating the SCN were directly photosensitive and able to convert electromagnetic radiation into neural signals. Melanopsin, a photopigment based on vitamin A, was found in these RGCs and is the strongest candidate for the circadian photopigment within these cells [7]. Genetically-manipulated mice that do not have melanopsin still show phase shifting by light exposure, although to a lesser degree [8]. This result, as well as more recent data from Hattar et al. [9], Panda et al. [10] and from Bullough et al. [11] seem to demonstrate that classical photoreceptors (rods and cones) as well as melanopsin-expressing RGCs participate in circadian phototransduction of mammals.

The spectral sensitivity of the human circadian system peaks between 440 and 500 nm [12,13]. Those data [12,13] are consistent with the conclusion that, overall, human melatonin suppression is dominated by at least two (not just one) opsins. However, the two studies [12,13] were conducted at similar times of the night, making it impossible to ascertain whether the spectral sensitivity of the circadian system changes at night. Two studies conducted in our own laboratory suggest that this might be true. Human adult males were exposed to a combination of two light levels and two broadband spectral power distributions (SPDs) from fluorescent lamps every two hours (at 00:00, 02:00, 04:00 and 06:00) for four nights in a counterbalanced order [14,15]. The results suggested that the spectral sensitivity of melatonin suppression may *change *during the night, because the relative contribution of the candidate photopigments (traditional photoreceptors and melanopsin-expressing RGCs) to best fit the suppression data seemed to systematically change during the night. The data obtained from broadband fluorescent light were not sufficiently precise, however, to determine which of several possible combinations of retinal photopigments participated in the circadian response to light.

In addition to the studies of the circadian system's response to light, and perhaps of direct relevance, several studies have shown that the absolute sensitivity of the visual system changes over the course of the night [16-18]. Increment thresholds to visual targets are apparently lowest just before dark and highest just before dawn [18]. Dacey et al. [19] have recently shown that in macaque (and, therefore, probably in humans as well), photosensitive melanopsin-expressing RGCs have input to the lateral geniculate nucleus (LGN), a major neural relay station from the retina to the visual cortex. If the overall sensitivity to light increases over the course of the night in this newly discovered class of RGCs, two results could occur. First, these cells could, in effect, set a higher luminous background on which a visual target must be detected, thus, increasing increment thresholds in the early morning relative to the early night. Second, the spectral sensitivity of the visual and circadian systems could shift to shorter wavelengths as the melanopsin-expressing RGCs become more dominant because their peak spectral response is at or near 480 nm.

Although a change in absolute sensitivity of the visual system over the course of the 24-hour day has been studied, there are no comparable studies for a change in the absolute sensitivity of the circadian phototransduction system. In part at least, this may be the result of the inherent nature of the outcome measures used in most studies of the circadian system. Changes in nocturnal melatonin production, core body temperature and phase shifting, the most common outcome measures used to evaluate the circadian system's response to light, can be the result of changes in the circadian phototransduction mechanism in the retina, the circadian clock in the SCN, or both. Changes in the relative values of these outcome measures to two different lights at two different times of night could, however, indicate a change in the circadian phototransduction mechanism. The experiment reported here was designed to investigate, using the relative difference in melatonin suppression and iris construction by two different light spectra, whether the spectral sensitivity of the circadian system changes at two different times of the night and thereby determine whether there was evidence for a temporal change in the retinal circadian phototransduction mechanism. The data used as the basis for this report are the same as those previously published suggesting spectral opponency in the human circadian phototransduction system [20,21].

## Methods

**Figure 1:**
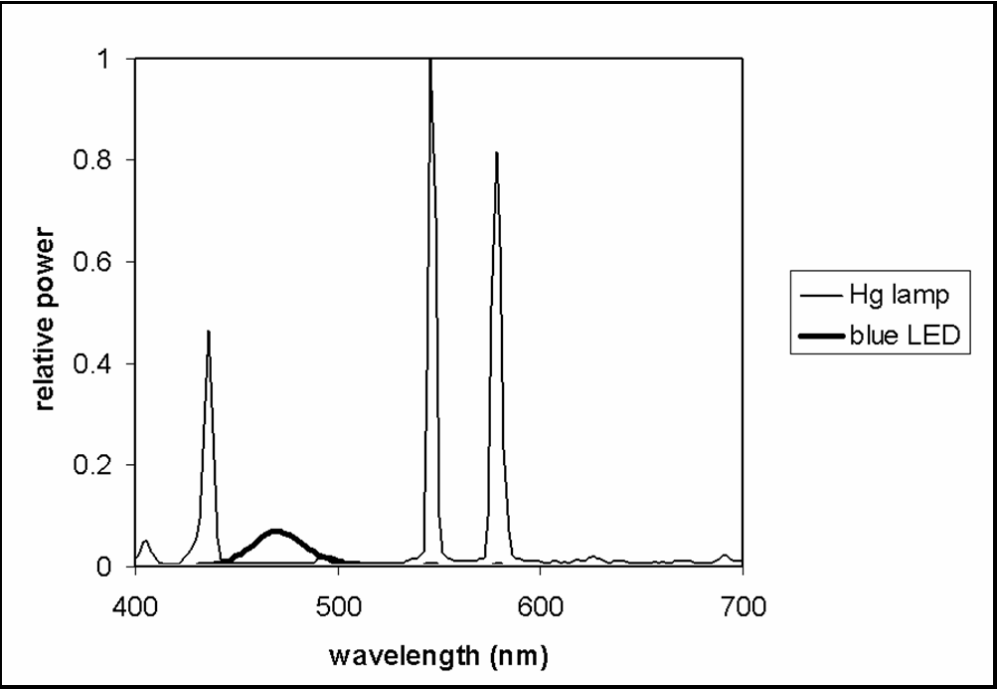
Relative spectral power distributions of the light sources used in the experiment.

Both melatonin suppression and iris constriction were measured during and after a one-hour light exposure just after midnight and just before dawn. A clear, high-pressure mercury (Hg) lamp and an array of blue (λ_max _= 470 nm) light emitting diodes (LEDs) were used (Figure 1). The Hg lamp provided 450 lx (170 μW/cm^2^) at the cornea and the set of LEDs provided 18 lx (29 μW/cm^2^) at the cornea. These light sources and light levels were selected to ensure that the suppression of melatonin for either light source was not high enough to produce asymptotic melatonin suppression [21].

Four male subjects, 20 or 21 years of age, participated in the study during two nights in May 2003. Each session lasted 8.5 h (from 22:30 to 07:00 h). All subjects signed a consent form approved by Rensselaer's Institute Review Board (IRB).

Each subject was seated in front of a 0.6 × 0.6 × 0.6 m plywood and matte-white painted box resting atop a small table, 0.76 m above the floor. The fronts of the boxes contained square 0.45 × 0.45 m apertures and chin rests so that every subject's face was inside one of the boxes. The backs of the boxes also had a square 0.3 × 0.3 m aperture behind which a computer monitor was placed. The computer monitors were adjusted so that only the red phosphor was used and provided no more than 3 lx at subjects' eyes when they sat at the boxes. Another small hole in the back of each box accommodated the zoom lens of a digital video camera, which was used to measure pupil size, as descried below.

The roofs of two boxes supported an uncoated, 175 W high-pressure Hg lamp (General Electric HR175A39) and ballast. When energized, the Hg lamps provided diffuse illumination throughout the box; light levels were controlled with mechanical filters and a neutral density acrylic filter (25% transmission). The inside front faces of the other two boxes were lined with an array of blue LEDs (Color Kinetics iCove) which provided diffuse illumination throughout the box; light levels were controlled electronically. As previously stated, each Hg lamp provided 450 lx (170 μW/cm^2^) at the cornea when a subject was seated at the table supporting the box and positioned in the chin rest; the set of LEDs provided 18 lx (29 μW/cm^2^) at the cornea.

All subjects followed their normal routine but refrained from consuming caffeinated products for 12 h before each session. Upon arrival at the facility, a registered nurse inserted a catheter into an arm vein of each subject. At 23:30, the first session of the night began by extinguishing all light in the laboratory except that from two red LED traffic signal lights that provided dim (<3 lx at the eye) ambient illumination throughout the laboratory. At midnight subjects were assigned to a light box for the entire night and asked to sit in front of it while wearing dark glasses, and before the Hg and LED light sources were energized. Subjects that were assigned to an Hg-illuminated box on the first night were assigned to an LED-illuminated box on the second night, and vice versa. During this time and throughout the night, subjects could interact with the modified computer monitor by playing video games or corresponding with friends on the Internet while their heads were positioned in the chin rest.

During the first session, three sets of three blood samples (3 ml each) were collected in the dark every 15 min., starting at 00:30. At 01:00 the light sources were energized, and four sets of three blood samples were collected every 15 min. from every subject until 02:00, at which time the lights were either extinguished or the subjects were asked to close their eyes. Three more sets of three blood samples were collected in the dark every 15 min. until 02:45.

Because the catheter was flushed with saline each time before blood samples were collected, the first blood sample collected in every set was always discarded. The two remaining samples in each set were immediately spun in a centrifuge at 3200 rpm (approximately 1000 × g) to obtain the plasma, which was then frozen at -85°C. Frozen samples were subsequently sent to an independent laboratory (Neuroscience Inc., Osceola, WI) for melatonin radioimmunoassay (Melatonin^direct ^I-125 RIA). The limit of detection of the assay was 1.5 pg/ml. The intra assay coefficients of variation (CVs) were 12.1% at 16.5 pg/ml, 5.7% at 68.7 pg/ml, and 9.8% at 162.7 pg/ml. The inter assay CVs were 13.2% at 17.3 pg/ml, 8.4% at 69 pg/ml, and 9.2% at 164.7 pg/ml.

Between blood sample collections, the irises of the subject were videotaped for one minute twice in the dark prior to light exposure, three times during light exposure, and, again, twice in the dark following the light exposure. During videotaping, subjects looked at a fixation point on the computer monitor so that pupil size did not vary with accommodation. Subsequently, images were digitized and pupil sizes were measured. After the experiment was completed, a video-editing program (Adobe Premiere 6.0) was used to capture six video images every 10 s from each subject at each experimental condition. If the subject blinked at the moment of video-frame capture, the video capture was sampled just before or just after the blink occurred. These captured images were then used for the pupil measurements. Pupil measurements were performed using MatLab 6.5. This program's unit of measure was pixels and pupil measurements were based on the relationship between the pupil and iris area because the position of the eye was not constant among the subjects. For each of the six images captured, three measurements of the pupil diameter and three measurements of the iris diameter were taken. It was assumed that a circle could mathematically represent both the pupil and iris. A relative measurement, referred to as relative pupil area (rPA) was obtained by dividing the pupil area by the iris area. Only the rPA values obtained during the light exposure periods were analyzed. All of the rPA values for a one-minute recording session were averaged to give a single estimate of pupil size during that period. Thus, three estimates of pupil size were obtained for both lighting conditions for every subject on both nights.

At 04:00, session two of the night began by asking subjects to again be seated in front of their assigned boxes. As before, ten blood samples were collected every 15 minutes and subjects' irises were videotaped between blood sample collections. After completion of the second session, the catheters were removed. Subjects left the laboratory at 07:00.

## Results

**Figure 2:**
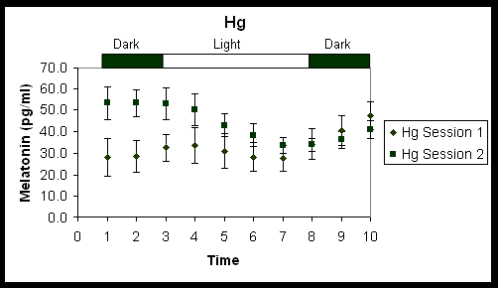
Average melatonin concentrations (pg/ml ± S.E.M.) under the Hg lighting condition.

**Figure 3:**
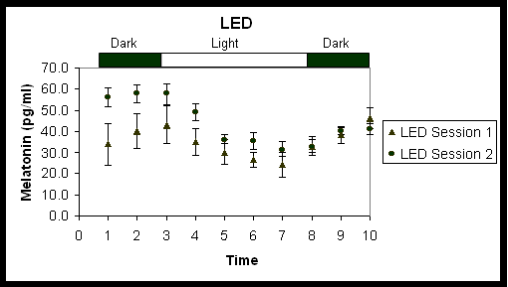
Average melatonin concentrations (pg/ml ± S.E.M.) under the LED lighting condition.

**Figure 4:**
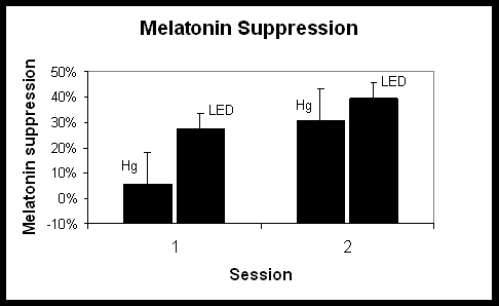
Melatonin suppression (mean ± S.E.M.) for sessions 1 and 2 for each lighting condition (Hg and LED).

Figures 2 and 3 show the average melatonin concentrations (pg/ml) under each combination of session and lighting conditions. Melatonin concentrations were totaled so that an overall suppression of melatonin for each combination could be determined. Average melatonin suppression (in percent) and standard error of the mean (S.E.M.) under Hg and LED lighting conditions for each session were then calculated using the melatonin concentrations from the last two measurements before light onset and melatonin concentrations from the last two measurements before light offset (Figure 4). A repeated-measures analysis of variance (ANOVA) showed a significant main effect for lighting condition (*F*_1,7 _= 8.48, p = 0.02). Overall, melatonin suppression for the LED condition was significantly greater than it was for the Hg lighting condition. The main effect for time of night was not significant (*F*_1,7 _= 4.71, p = 0.07) although melatonin levels during the dark periods prior to any light exposure were higher in session 2 than in session 1 (Figures 2 and 3). Post-hoc statistical tests were conducted to determine whether there was a significant change in melatonin suppression from session 1 to session 2 for each lighting condition, LED and Hg (Figure 4). A paired, one-tailed Student's t-test showed significantly more suppression of melatonin in session 2 than in session 1 for the Hg lighting condition (*t*_7 _= 3.11, p = 0.008), but there was no significant difference in melatonin suppression for the LED lighting condition at the two times of night (*t*_7 _= 0.96, p = 0.2). Mann-Whitney nonparametric statistical tests were also conducted and revealed similar results as the parametric tests (i.e., significant main effect of lighting condition, but not of session time).

**Figure 5:**
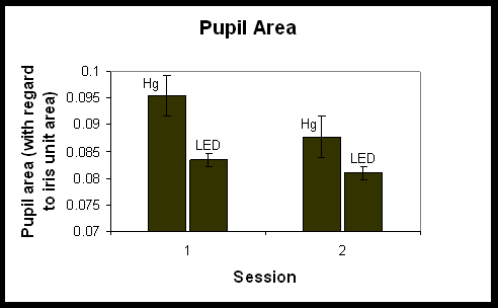
Relative pupil area (calculated as a proportion of the iris area, normalized to unity, ± S.E.M.) for sessions 1 and 2 for each lighting condition (Hg and LED).

The pupil size results showed similar trends, but in the opposite direction. Figure 5 shows the rPA (calculated as described above as the proportion of the iris area, normalized to unity) for the Hg and LED lighting conditions in sessions 1 and 2. A repeated-measures ANOVA showed a significant main effect for lighting condition (*F*_1,5 _= 12.63, p = 0.02). Overall, pupil size for the LED condition was significantly smaller than it was for the Hg lighting condition. Overall, pupil sizes were larger in session 1 than in session 2 (Figure 5). Post-hoc statistical tests were conducted to determine whether there was a significant change in pupil size from session 1 to session 2 for each lighting condition, LED and Hg. A paired, one-tailed Student's t-test showed that pupil area in session 1 was significantly larger than in session 2 for Hg (*t*_5 _= 1.96, p = 0.05), but there was no significant difference in pupil size for the LED lighting condition at the two times of night (*t*_5 _= 0.62, p = 0.3). These results suggest that the iris light reflex is less affected by the Hg lighting condition in session 1 (resulting in a larger pupil size) than in session 2. As with the melatonin suppression data, Mann-Whitney nonparametric statistical tests were conducted and revealed similar results.

It should further be noted that overall suppression of melatonin for the LED lighting condition was significantly higher than for the Hg lighting condition, even though the pupil areas for the LED lighting condition were smaller. Since pupil size is determined, in part, by the retinal exposure to light and, thus influences the amount of melatonin suppression [22], the difference between melatonin suppression by the Hg and LED spectra would have been relatively larger if pupil sizes were held constant throughout the experiment.

## Discussion

The increase in melatonin suppression and in iris constriction for the Hg source relative to the LED source at night suggests a temporal change in the photoreceptor mechanisms contributing to the circadian system phototransduction. Although a discussion of the recently published model of human circadian phototransduction by Rea et al. [23] is beyond the scope of this short communication, the model does predict greater overall melatonin suppression from the blue LED source at 18 lx (29 μW/cm^2^) at the cornea than from the Hg source at 450 lx (170 μW/cm^2^) at the cornea. The model, based upon retinal neuroanatomy and electrophysiology, incorporates input from conventional photoreceptors and from melanopsin-expressing RGCs to predict the circadian light stimulus from both monochromatic and polychromatic light sources. It does not, however, make provision for a change in the spectral sensitivity of circadian phototransduction at different times of the night. The model could accommodate a change in spectral sensitivity through a temporally dependent coefficient modulating the relative magnitude of the contribution to the overall spectral sensitivity by the melanopsin-expressing RGCs. Indeed, to model an increasing contribution of the melanopsin-expressing RGCs at different times of the night, we increased the value of that coefficient and found that the Hg and LED sources would have much closer predicted circadian stimulus values and would, thus, produce similar levels of melatonin suppression. In other words, a simple increase in the relative contribution of the melanopsin-expressing RGCs near morning would account for the smaller difference in melatonin suppression between the Hg and the LED conditions in session 2 (between 04:00 and 05:00) than the difference between those two lighting conditions in session 1 (between 01:00 and 02:00). It should be noted that the model by Rea et al. [23] does not deal quantitatively with circadian input to the light reflex of the iris, including a possible change in the spectral sensitivity of the iris light reflex at night.

Two observations might initially be offered in contradiction to the inference that the spectral sensitivity of the circadian system changes at night. First, the difference in relative suppression might be an artifact of having different amounts of melatonin to suppress throughout the night (i.e., there was more melatonin to suppress later at night than early in the night). Under no experimental condition, however, was light intensity strong enough to suppress melatonin to daytime levels (below 12 pg/ml). Since there was always melatonin to be suppressed by light, the percent melatonin suppression should be unrelated to the absolute level of melatonin available. Certainly, percent suppression is accepted in the literature as a measure of the impact of light on the circadian system [12-15]. Given enough melatonin to suppress under all lighting conditions, the differential effects of the two spectra at the two times of the night strongly suggest that there is a change in the spectral sensitivity of the retinal phototransduction mechanisms of the circadian system. Second, the absolute reduction in pupil size at night might be the result of increased fatigue [24-26]. This interpretation is also likely incomplete because, again, of the relative impact on pupil constriction by the two sources at the two times of the night and because neither lighting condition produced maximum constriction of the pupil. Moreover, pupil constriction mirrored the percentage of melatonin suppression for the two sources over time, indicating similar underlying phototransduction mechanism. In this context, it should be recalled that Lucas et al. [5] showed that in mice, pupil constriction, as well as phase shifting, is influenced by melanopsin-expressing, intrinsically photosensitive RGCs. Nevertheless, the pupil size data presented here should be interpreted with caution. Human pupil size is notoriously variable [27,28], especially because the balance between sympathetic and parasympathetic input to the iris response can vary moment to moment, at different times of day, and for different tasks [24-26].

These data suggest, despite inherent uncertainty in the pupil size measurements, that the mirrored changes in pupil constriction and melatonin suppression reflect changes in the relative photoreceptor contributions to the circadian phototransduction system at night and that these changes could be related to increased participation by melanopsin-expressing RGCs closer to the morning. As discussed in the introduction, the earlier data by Rea et al. [14,15] are consistent with this interpretation as, at least indirectly, are some recent evidence from Hannibal et al. [29] suggesting that gene expression of melanopsin in the photosensitive RGCs of the albino Wistar rat follow a circadian pattern. Finally, these findings might provide additional insight into the reported changes in visual thresholds at night [18].

## Conclusion

The results presented here are the first to suggest a temporal change in spectral sensitivity of the human circadian system phototransduction at two different times during the night, measured through nocturnal melatonin suppression, and with less certainty (owing to the inherent variability of the iris constriction response) through pupil area.

## Competing interests

The author(s) declare that they have no competing interests.

## Authors' contributions

**MGF **helped with the conception and design of the experiment, collected the data, participated in the data analyses and interpretation, and helped to draft the manuscript.

**JDB **helped with the conception and design of the experiment, helped to collect the data, participated in the data analyses and interpretation, and helped to draft the manuscript.

**RHP **helped with the conception and design of the experiment and participated in the data analyses and interpretation.

**MSR **conceived the study, helped to collect the data, participated in the data analyses and interpretation, and helped to draft the manuscript.

All authors read and approved the final manuscript.
